# The Effects of Tau, Amyloid, and White Matter Lesions on Mobility, Dual Tasking, and Balance in Older People

**DOI:** 10.1093/gerona/glaa143

**Published:** 2020-06-07

**Authors:** Maria H Nilsson, Gro Gujord Tangen, Sebastian Palmqvist, Danielle van Westen, Niklas Mattsson-Carlgren, Erik Stomrud, Oskar Hansson

**Affiliations:** 1 Department of Health Sciences, Faculty of Medicine, Lund University, Sweden; 2 Clinical Memory Research Unit, Faculty of Medicine, Lund University, Sweden; 3 Memory Clinic, Skåne University Hospital, Malmö, Sweden; 4 Norwegian National Advisory Unit on Ageing and Health, Vestfold Hospital Trust, Tonsberg, Norway; 5 Department of Geriatric Medicine, Oslo University Hospital, Norway; 6 Diagnostic Radiology, Department of Clinical Sciences, Lund University, Sweden; 7 Image and Function, Skane University Hospital, Lund, Sweden; 8 Wallenberg Center for Molecular Medicine, Lund University, Sweden

**Keywords:** Biomarkers, Dementia, Magnetic resonance imaging, Postural balance, Difficulty walking

## Abstract

**Background:**

This study aimed to investigate whether white matter lesions (WML), β-amyloid-, and tau pathologies are independently associated with mobility, dual tasking, and dynamic balance performance in older nondemented individuals.

**Methods:**

We included 299 older people (mean, *SD*, age: 71.8, 5.6 years) from the Swedish BioFINDER study, whereof 175 were cognitively unimpaired and 124 had mild cognitive impairment (MCI). In multivariable regression analyses, dependent variables included mobility (Timed Up & Go [TUG]), dual tasking (TUG with a simultaneous subtraction task, that is, TUG-Cog, as well as dual task cost), and balance (Figure-of-eight). The analyses were controlled for age, sex, education, diagnosis (ie, MCI), and comorbidity (stroke, diabetes, and ischemic heart disease). Independent variables included WML volume, and measures of β-amyloid (abnormal cerebrospinal fluid [CSF] Aβ42/40 ratio) and tau pathology (CSF phosphorylated tau [p-tau]).

**Results:**

Multivariable regression analyses showed that an increased WML volume was independently associated with decreased mobility, that is, TUG (standardized *β* = 0.247; *p* < .001). Tau pathology was independently associated with dual tasking both when using the raw data of TUG-Cog (*β* = 0.224; *p* = .003) and the dual-task cost (*β*= −0.246; *p* = .001). Amyloid pathology was associated with decreased balance, that is, Figure-of-eight (*β* = 0.172; *p* = .028). The independent effects of WML and tau pathology were mainly observed in those with MCI, which was not the case for the effects of amyloid pathology on balance.

**Conclusions:**

Common brain pathologies have different effects where WML are independently associated with mobility, tau pathology has the strongest effect on dual tasking, and amyloid pathology seems to be independently associated with balance. Although these novel findings need to be confirmed in longitudinal studies, they suggest that different brain pathologies have different effects on mobility, balance, and dual-tasking in older nondemented individuals.

Decreased gait speed precedes cognitive decline among older people without dementia, and those who over time decline in both gait speed and cognition are more prone to progress to a major neurocognitive disorder, that is, dementia (1–4). To investigate how different brain pathologies relate to motor aspects prior to dementia is therefore of interest. Common brain pathologies in older people include cerebral small vessel disease (especially white matter lesions [WML]) (5), and aggregation of β-amyloid (Aβ) and tau. WML as well as Aβ and tau aggregates can be reliably detected in vivo (5,6).

Alzheimer’s disease (AD) is the most common cause of dementia among older adults. Aβ pathology is a sign of AD, which can be measured using the cerebrospinal fluid (CSF) Aβ42/40 ratio or by using positron emission tomography (PET), that is, amyloid-PET, which have high agreement (6,7). Tau pathology is the second neuropathological hallmark of AD, which can be measured using either CSF phosphorylated tau (p-tau) or tau-PET (8). Small vessel disease is detected and quantified using magnetic resonance imaging (MRI) (5). In AD, widespread amyloid pathology develops one to three decade before symptom onset and is not on its own strongly associated with cognitive impairment (9). Widespread neocortical tau pathology occurs later in the disease and is associated with development of MCI and dementia (10–12). When it comes to WML, such brain changes have been shown to be associated with both cognitive decline and worse motor performance in older people, including balance impairments and falls (13).

Previous PET studies have shown an association between Aβ burden and decreased gait performance in older people without dementia (14–18), but none of these addressed tau pathology. Recent studies showed that tau pathology is more associated with cognitive impairment than WML or Aβ pathology (12,19,20), which highlights the importance of also addressing tau pathology. Although gait and mobility are related, they are not interchangeable constructs, which also applies for balance performance. Importantly, previous studies (17,18,21–28) that addressed mobility and balance did not simultaneously consider WML, Aβ-, and tau pathology. Such knowledge is needed to gain an increased understanding of their independent effects. One way of assessing mobility is by using the Timed Up & Go (TUG) test (29). In studies that used TUG, WML detected by MRI were associated with decreased mobility in older people without dementia (21,22). PET studies that focused on Aβ and mobility (ie, TUG) showed conflicting results (18,24). A recent study that used CSF Aβ42 showed no significant correlation with mobility; nor did they find an association between CSF p-tau and mobility (25). In studies that focused on balance impairments, WML were associated with both balance impairments and falls (26–28). A PET study showed an association between Aβ pathology and declined static balance time in standing among cognitively unimpaired older persons (17). There is limited knowledge of how tau pathology relates to mobility and balance performance in older people without dementia, and studies are needed that include WML as well as Aβ- and tau pathology.

The dual-task paradigm can reveal subtle motor impairments that cannot be detected during single-task test conditions. When interested in the cognitive-motor interference, the dual-task paradigm involves a motor task and a concurrent cognitive task (30). Two studies reported that WML were associated with slower dual task gait speed in older people without cognitive complaints (31,32), but another study reported contradictory results (21). None of these studies addressed Aβ- and tau pathology. There is limited and conflicting evidence on how CSF biomarkers (Aβ and tau) relate to dual tasking (25,33). The latter two studies had relatively small sample sizes (*N* ≤ 90), and they included persons with dementia. Importantly, they did not simultaneously take into account different CSF biomarkers by using multivariable analyses; nor did they include WML (25,33). This warrants further studies with larger sample sizes that can identify independent effects.

This study aimed to investigate whether WML, amyloid-, and tau pathology are independently associated with mobility, dual tasking, and dynamic balance performance in older nondemented people with and without MCI. Amyloid pathology was determined with the CSF Aβ42/40 ratio, and tau pathology was measured with CSF p-tau in agreement with the research framework of the U.S. National Institute on Aging–Alzheimer’s Association guidelines (34).

Our a priori hypotheses were that WML are independently associated with mobility and balance whereas tau pathology is independently associated with decreased dual-task performance. The latter hypothesis is based on that tau pathology is more associated with cognitive impairment than WML or Aβ pathology (12,19,20), and we will investigate dual-task performance that address cognitive–motor interference. In relation to amyloid pathology, we had a hypothesis-free approach.

## Method

This cross-sectional study is part of the larger longitudinal Swedish BioFINDER study (www.biofinder.se). In the current study, participants were included if they had undergone a physical therapy assessment that addressed mobility, dual tasking, and balance. These assessments were done in 299 participants out of 591 in the larger BioFINDER study (Malmö, Sweden).

### Participants

The study population included 77 cognitively normal older individuals (recruited from the population-based Malmö Diet Cancer Study (35)) and 222 nondemented patients with cognitive complaints enrolled consecutively at one memory outpatient clinic in Sweden.

Inclusion criteria for cognitively normal older people were (i) age ≥60 y, (ii) Mini-Mental State Examination (MMSE) 28–30 points at the screening visit, (iii) absence of cognitive symptoms as evaluated by a physician, (iv) not fulfilling the criteria of MCI or any dementia disorder, and (v) fluent in Swedish. Exclusion criteria included the following: (i) significant neurological or psychiatric disease (eg, stroke, Parkinson disease, multiple sclerosis, and major depression), (ii) significant systemic illness making participation difficult, or (iii) significant alcohol or substance abuse.

The inclusion criteria for patients with cognitive complaints were (i) cognitive symptoms, (ii) age 60–80 y, (iii), MMSE 24–30 points, (iv) not fulfilling the criteria for any dementia disorder, and (v) fluent in Swedish. The exclusion criteria were (i) cognitive impairment that without doubt could be explained by another condition (other than prodromal dementia), (ii) significant systemic illness making participation difficult, or (iii) significant alcohol or substance abuse. This rendered a sample of 222 nondemented people with cognitive complaints. They were classified as having either subjective cognitive decline (SCD, *n* = 98) or MCI (*n* = 124, of which 90 [73%] had amnestic MCI and 34 [27%] had nonamnestic MCI). The classification was based both on a neuropsychological battery (the following cognitive domains were included: verbal ability, visuospatial construction, episodic memory, and executive functions) and the clinical assessment of a senior neuropsychologist. In agreement with U.S. National Institute on Aging–Alzheimer’s Association guidelines, persons who were cognitively normal and those with SCD were included in the cognitively unimpaired group (34), resulting in 175 cognitively unimpaired participants and 124 participants with MCI. Participants’ characteristics are presented in Table 1. For the total sample (*N* = 299), the mean MMSE score was 27.9 (*SD* 1.8). The corresponding mean values for those who were cognitively unimpaired were 28.7 (*SD* 1.3) versus 26.8 (*SD* 1.8) for those with MCI.

**Table 1.** Participants’ Characteristics and Missing Data, *N* = 299

	Total Sample		Cognitively Unimpaired		Mild Cognitive Impairment	
	*N* = 299	Miss, *n*	*n* = 175	Miss, *n*	*n* = 124	Miss, *n*
Age (y), mean (*SD*)	71.8 (5.6)	–	72.5 (5.6)	–	70.9 (5.4)	–
Sex (women), *n* (%)	142 (47.5)	–	86 (49.1)	–	56 (45.2)	–
Education (y), mean (*SD*)	11.8 (3.3)	3	12.2 (3.4)	–	11.1 (3.1)	3
Comorbidity: stroke/diabetes/heart disease (yes), *n*	27/32/39	0/1/1	10/19/17	–	17/13/22	0/1/1
Timed Up & Go, ie, TUG, (s), mean (*SD*)	11.6 (3.9)	4	11.0 (2.8)	2	12.4 (4.9)	2
TUG-Cog (s), mean (*SD*)	21.9 (18.9)	14*	18.3 (10.6)	5	27.2 (26.0)	9
Dual task cost (%), mean (*SD*)	−90.2 (145.8)	15	−67.9 (82.0)	6	−123.0 (202.7)	9
Figure-of-eight (s), mean (*SD*)	19.2 (10.5)	4^†^	17.6 (6.9)	1	21.5 (13.9)	3
WML (total volume, mL), mean (*SD*)	18.0 (21.9)	22	14 (16.8)	10	23.7 (26.8)	12
p-tau (ng/L), mean (*SD*)	58.8 (23.9)	20	54.5 (20.6)	16	64.7 (26.6)	4
CSF Aβ42/40 (pathological, <0.878), *n* (%)	111 (40.1)	22	42 (26.4)	16	69 (58.5)	6

*Notes:* Miss, missing values; WML, white matter lesions; p-tau, phosphorylated tau; Aβ, β amyloid. TUG-Cog, ie, TUG with a simultaneous subtraction task.

*10 out of these 14 did not manage TUG-Cog, whereof 6 had MCI.

^†^Two did not manage the Figure-of-eight.

### Standard Protocol Approvals, Registrations, and Patient Consents

The study was approved by the Regional Ethics Committee in Lund (2008/695 and 2010/296), Sweden, and all study participants gave their written informed consent.

### Assessments by the Physical Therapist

The TUG assesses functional mobility and includes the following tasks: to rise from a chair, walk 3 m, turn around, and walk back to sit down again (29). The participant’s regular footwear was used and customary walking aid, but no physical assistance was given. Two trials were conducted: (i) TUG in comfortable gait speed and (ii) TUG in comfortable gait speed with a simultaneous serial subtraction task by 3 (starting: 100–3), that is, TUG-Cog. No instruction was given whether the participant should prioritize the motor and/or the cognitive task. The time (s) to complete each TUG trial was registered. Timing commenced when the participant’s back was leaving the back of the chair and stopped when the buttock reached the seat of the chair. For each individual, the dual-task cost (%) was calculated by using the recommended formula (36): ([simple-task gait value − dual-task gait value]/simple-task gait value) × 100, that is, ([TUG–TUG-Cog]/TUG) × 100. In this study, the dual-task cost value (%) reflects how an increased cognitive load affects mobility. Negative values reflect that mobility is negatively influenced by adding the cognitive task.

The Figure-of-eight was developed as a balance task; it is striped to the floor as two circles that are open in the intersection (37,38). In the current study, each inner circle had a diameter of 157 cm. Outer lines were striped, which created a walking path of 10 cm. The participant initiated the test at the top (facing a counterclockwise direction), and the instruction was to step within the two stripes and complete walking the eight. No walking aids were allowed; five participants were therefore excluded in this test. One trial was conducted. The total time for completion was registered as well as number of oversteps, that is, steps where half of the foot was placed outside the lines. The total time of the Figure-of-eight was chosen as the dependent variable since it is more reliable than oversteps (38).

The test order was as follows: TUG (29); TUG-Cog; and Figure-of-eight (37,38). Two experienced physical therapists conducted the assessments, and all assessments were video recorded according to a standardized protocol. To eliminate minor discrepancies between the two assessors, the current ratings and timing were based on the video recordings retrospectively. A digital stopwatch was used when timing the tasks.

### CSF Sampling/Analysis and MRI

Lumbar puncture and CSF handling followed a structured protocol as previously described (39). CSF Aβ42 and Aβ40 were analyzed using Euroimmun ELISAs (EUROIMMUN AG, Lübeck, Germany) and p-tau181 was analyzed using Innotest ELISA (Fujirebio Gent, Belgium). Due to the biomodal distribution the CSF Aβ42/Aβ40 ratio mixture modeling was used to identify an unbiased cutoff for abnormal Aβ accumulation (<0.878), as previously described (40,41).

MR imaging was performed at the same Siemens Trio 3 T system in all individuals and included transversal T2 FLAIR and a high-resolution isotropic MPRAGE. Automated segmentation of WML using the LST toolbox implemented in SPM8, generated a total lesion volume (mL), here named “WML volume,” for each individual (42).

### Statistical Analyses

Pearson (*r*) and Spearman’s correlation coefficients (*r*_s_) were used to study the relationships among the independent variables in order to detect any multicollinearity. The following independent variables were considered: WML volume, CSF p-tau, and CSF Aβ42/40 (1 = abnormal, <0.878).

Associations between each dependent variable and independent variables (WML, CSF p-Tau, and CSF Aβ42/40) were analyzed in a series of univariable regression models. Linear regression analyses were used for all dependent variables: TUG, TUG-Cog, dual-task cost, and for the total time of the Figure-of-eight. The simple regression analyses are presented in Supplementary File S1.

For the total sample, this was followed by multivariable regression analyses that included only one brain pathology, including controlling for age (y), sex (1 = woman), education (y), sample (1 = MCI), and comorbidity (Table 2). Data on comorbidity included dichotomous (No/Yes) variables in relation to stroke, diabetes, and ischemic heart disease.

**Table 2.** Multivariable Analyses (Total Sample); Each Controlled for Age, Sex, Education, Diagnosis, and Comorbidity

DV	Mobility	Dual Tasking		Balance
	TUG (s)	TUG-Cog (s)	Dual Task Cost (%)	Figure-of-Eight (s)
Independent variables	B (95% CI) *β*; *p* value	B (95% CI) *β*; *p* value	B (95% CI) *β*; *p* value	B (95% CI) *β*; *p* value
1. WML volume, mL	0.040 (0.020, 0.061) 0.249; **<.001**	0.090 (−0.010, 0.190) 0.117; .078	−0.155 (−0.944, 0.635) −0.026; .700	0.034 (−0.033, 0.100) 0.066; .319
2. p-tau, ng/L	−0.015 (−0.033, 0.004) −0.099; .119	0.145 (0.049, 0.242) 0.186; **.003**	−1.38 (−2.16, −0.591) −0.219; **.001**	0.041 (−0.016, 0.098) 0.089; .162
3. CSF Aβ42/40 (1 = abnormal)	−0.182 (−1.09, 0.727) −0.026; .693	3.95 (−0.821, 8.71) 0.105; .104	−39.9 (−78.6, −1.24) −0.134; **.043**	3.62 (0.830, 6.41) 0.164; **.011**

*Notes:* DV, dependent variables; TUG, Timed Up & Go; TUG-Cog, TUG with a simultaneous subtraction task; WML, White matter lesions; p-tau, phosphorylated tau; CSF, cerebrospinal fluid; Aβ, β amyloid. Multivariable analyses (1–3) included one brain pathology, ie, WML, p-tau, or CSF Aβ42/40; controlled for age, sex, education, diagnosis, and comorbidity. For dichotomous independent variables: 0, reference category. Dual task cost (%): ([TUG–TUG-Cog]/TUG) × 100. See Supplementary Table S1 for simple regression analyses.

In the following step, the multivariable analyses included controlling factors (ie, age, sex, education, sample, and comorbidity) and different brain pathologies (ie, WML volume, CSF p-tau, and CSF Aβ42/40), which were simultaneously entered (Table 3). That is, independent variables (ie, brain pathologies) were included regardless of their *p* value in the simple regression analyses, that is, in order to not leave out a potential confounding factor. The models were not reduced (ie, manually removing variables based on their *p* value) since the aim was not to provide prediction models but to gain an increased understanding of the independent association between pathology and mobility, dual tasking and balance, respectively. For multivariable linear regression analyses, residuals were inspected graphically as a validation of each model.

**Table 3.** Multivariable Analyses (Total Sample); Controlled for Different Brain Pathologies, Age, Sex, Education, Diagnosis, and Comorbidity

DV	Mobility	Dual Tasking		Balance
	TUG (s) *n* = 251	TUG-Cog (s) *n* = 240	Dual Task Cost (%) *n* = 240	Figure-of-Eight (s) *n* = 247
	B (95% CI) *β*; *p* value	B (95% CI) *β*; *p* value	B (95% CI) *β*; *p* value	B (95% CI) *β*; *p* value
WML volume, mL	0.039 (0.018, 0.060) 0.247; **<.001**	0.081 (−0.016, 0.179) 0.109; .103	−0.121 (−0.912, 0.670) −0.021; .763	0.050 (−0.020, 0.121) 0.096; .161
p-tau, ng/L	−0.016 (−0.037, 0.006) −0.107; .151	0.152 (0.054, 0.250) 0.224; **.003**	−1.32 (−2.11, −0.532) −0.246; **.001**	0.004 (−0.066, 0.075) 0.009; .907
CSF Aβ42/40 (1 = abnormal)	0.493 (−0.543, 1.53) 0.071; .349	0.438 (−4.34, 5.21) 0.014; .857	−2.23 (−40.8, 36.3) −0.009; .909	3.86 (0.413, 7.30) 0.172; **.028**

*Notes:* DV, dependent variables; TUG, Timed Up & Go; TUG-Cog, TUG with a simultaneous subtraction task; WML, White matter lesions; p-tau, phosphorylated tau; CSF, cerebrospinal fluid; Aβ, β amyloid. For dichotomous independent variables: 0, reference category. Dual task cost (%): ([TUG − TUG-Cog]/TUG) × 100.

Multivariable analyses were rerun for those who were cognitively unimpaired or who had MCI, respectively (Tables 4–5). These analyses were controlled for age, sex, education, and comorbidity.

**Table 4.** Multivariable Regression Analyses with TUG or TUG-Cog as Dependent Variables in Cognitively Unimpaired and MCI, Respectively

DV	Mobility: Timed Up & Go (TUG)		TUG-Cog, ie, TUG + Subtraction Task	
	Cognitively Unimpaired* *n* = 149	MCI* *n* = 102	Cognitively Unimpaired* *n* = 145	MCI* *n* = 95
	B (95% CI) *β*; *p* value	B (95% CI) *β*; *p* value	B (95% CI) *β*; *p* value	B (95% CI) *β*; *p* value
WML volume, mL	0.013 (−0.017, 0.043) 0.081; .385	0.058 (0.027, 0.089) 0.365; ** <.001**	−0.008 (−0.096, 0.080) −0.017; .855	0.146 (−0.031, 0.323) 0.171; .105
p-tau, ng/L	0.005 (−0.022, 0.032) 0.039; .707	−0.034 (−0.067, <0.001) −0.208; .051	0.023 (−0.056, 0.103) 0.061; .559	0.281 (0.097, 0.466) 0.326; ** .003**
CSF Aβ42/40 (1 = abnormal)	0.376 (−0.822, 1.57) 0.062; .536	0.364 (−1.42, 2.15) 0.043; .686	0.647 (−2.85, 4.15) 0.037; .715	0.887 (−9.15, 10.92) 0.019; .861

*Notes:* DV, dependent variables; MCI, mild cognitive impairment; WML, white matter lesions; p-tau, phosphorylated tau; CSF, cerebrospinal fluid; Aβ, β amyloid.

*Controlled for different brain pathologies, age, sex, education, and comorbidity.

**Table 5.** Multivariable Regression Analyses with Dual Task Cost and Balance as the Dependent Variables in Cognitively Unimpaired and MCI, respectively

DV	Dual Task Cost (%)		Balance: Figure-of-Eight	
	Cognitively Unimpaired* *n* = 145	MCI* * n* = 95	Cognitively Unimpaired* *n* = 148	MCI* * n* = 99
	B (95% CI) *β*; *p* value	B (95% CI) *β*; *p* value	B (95% CI) *β*; *p* value	B (95% CI) *β*; *p* value
WML volume, mL	0.299 (−0.517, 1.11) 0.068; .470	−0.455 (−1.84, 0.928) −0.068; .515	0.015 (−0.062, 0.091) 0.034; .706	0.090 (−0.038, 0.218) 0.157; .164
p-tau, ng/L	−0.159 (−0.893, 0.575) −0.045; .668	−2.45 (−3.89, −1.01) −0.366; **.001**	−0.005 (−0.073, 0.063) −0.014; .892	0.005 (−0.131, 0.142) 0.009; .938
CSF Aβ42/40 (1 = abnormal)	0.719 (−31.6, 33.1) 0.004; .965	−13.67 (−92.08, 64.75) −0.039; .730	2.80 (−0.261, 5.86) 0.176; .073	4.60 (−2.60, 11.8) 0.154; .207

*Notes:* DV, dependent variables; MCI, mild cognitive impairment; TUG, Timed Up & Go; TUG-Cog, TUG with a simultaneous subtraction task; WML, white matter lesions; p-tau, phosphorylated tau; CSF, cerebrospinal fluid; Aβ, β amyloid. Dual task cost (%): ([TUG − TUG-Cog]/TUG) × 100.

*Controlled for different brain pathologies, age, sex, education, and comorbidity.

All statistical analyses were performed using SPSS Windows 24.0 (IBM SPSS Inc., Chicago, IL, USA).

## Results

Descriptive data and missing data are reported in Table 1, and simple regression analyses are presented in Supplementary File S1.

Multivariable regression analyses that included one brain pathology (controlling for age, sex, education, diagnosis, and comorbidity: stroke, diabetes, and ischemic heart disease) are presented in Table 2. Increased WML volume was the only variable significantly associated with mobility, that is, more WML were associated with an increased time to complete TUG (standardized beta, ie, *β* = 0.249; *p* < .001). Although WML volume was not significantly associated with dual tasking, there was a trend in relation to TUG-Cog (*β* = 0.117; *p* = .078) but not for the dual-task cost (*β* = −0.026; *p* = .700). Higher CSF p-tau was associated with dual tasking, which applied both for TUG-Cog (*β* = 0.186; *p* = .003) and the dual-task cost (*β* = −0.219; *p* = .001). An abnormal CSF Aβ42/40 ratio was associated with an increased dual-task cost by 40% (*β* = −0.134; *p* = .043), whereas Aβ was not associated with TUG-Cog (*p* = .104). In addition, the CSF Aβ42/40 ratio was associated with dynamic balance, that is, Figure-of-eight (*β* = 0.164; *p* = .011). This was not the case for WML volume (*p* = .319) or tau pathology (*p* = .162).

Multivariable analyses that involved the total sample (controlled for age, sex, education, diagnosis, and comorbidity) and simultaneously including WML, CSF p-tau, and CSF Aβ42/40 are presented in Table 3. Dependent variables were mobility (ie, TUG), dual tasking (TUG-Cog and dual-task cost, respectively), and balance (Figure-of-eight). WML volume was the only variable that was independently associated with mobility, that is, TUG (standardized beta, ie, *β* = 0.247; *p* < .001). CSF p-tau was the only variable independently associated with dual tasking, which applied both for the raw data of TUG-Cog (*β* = 0.224; *p* = .003) and the dual-task cost (*β* = −0.246; *p* = .001). An abnormal CSF Aβ42/40 ratio was the only variable significantly associated with decreased balance, that is, Figure-of-eight (*β* = 0.172; *p* = .028).

The independent effect of WML on mobility was significant among those with MCI (*β* = 0.365; *p* < .001), but it was not significant (*p* = .385) among those who were cognitively unimpaired (Table 4). The same pattern was shown in relation to the effect of tau pathology on dual tasking. CSF p-tau was independently associated with TUG-Cog (*β* = 0.326; *p* = .003) and the dual-task cost (*β*= −0.366; *p* = .001) among those with MCI, which was not the case (*p* ≥ .559) among those who were cognitively unimpaired (Tables 4–5). An abnormal CSF Aβ42/40 ratio was no longer significantly associated with balance, that is, among cognitively unimpaired (*β* = 0.176; *p* = .073) and those with MCI (*β* = 0.154; *p* = .207; Table 5).

## Discussion

This study provides novel knowledge by using multivariable analyses that take into account WML as well as markers of Aβ- and tau pathology in relation to mobility, dual tasking, and balance performance. This allowed studying independent effects of different common brain pathologies. In the total sample, we found that WML volume was independently associated with decreased mobility, and tau pathology was independently associated with dual tasking, whereas Aβ pathology was independently associated with dynamic balance in older persons without dementia. These findings might reflect the temporal evolution of Aβ- and tau pathology, where Aβ pathology precedes tau pathology by decades and that tau pathology is more associated with cognitive impairment than Aβ pathology (9–12,20).

The independent effects of WML and tau pathology were mainly observed in cases with MCI, which is interesting considering that these pathologies are associated with cognitive symptoms (12,19,20). On the contrary, amyloid pathology and balance tended to be associated in cognitively unimpaired, which makes sense considering that amyloid pathology develops about 20 y before MCI in AD (9).

### Mobility

In the total sample, the volume of WML was the only variable that was independently associated with decreased mobility (ie, TUG) with and without adjusting for other biomarkers. That WML are associated with decreased mobility is in line with two prior studies in older people without dementia (*N* = 201 and *N* = 171, respectively) (21,22), although a smaller (*N* = 80) study found no such association (23). The effect of WML on mobility predominated in the MCI group, that is, despite the fact that we had controlled for MCI when analyzing the total sample.

In the total sample, CSF Aβ or p-tau were not associated with mobility, which is in line with a recent study that used CSF measures (25). Although PET studies that focused on Aβ and mobility (ie, TUG) showed conflicting results (18,24), a study that adjusted for WML load (ie, Fazekas score) showed no association between Aβ and mobility (24). The current findings add to the body of knowledge since our analyses simultaneously considered WML, Aβ, and tau pathology, as well as other factors such as cognitive status.

It needs to be noted that TUG was developed to capture functional mobility (29), and the time to complete TUG does not mimic gait speed. Although TUG includes walking, it also includes rising and sitting down as well as turning. That is, studies that address gait speed are not directly comparable with those focusing on mobility by using TUG. Mobility (ie, as assessed by using TUG) and gait are not interchangeable constructs. This was recently emphasized in a review article that addressed neural correlates of gait, and they therefore excluded studies that used TUG as an outcome measure (43). A recent meta-analysis showed that decreased gait speed was associated with impairment in global cognition (44). We acknowledge that it would have been interesting to include also objective gait measures that tap different components of gait. Not the least as several PET studies have shown an association between amyloid burden and gait, for example, slow gait speed (14–18). In contrast to the current study, these prior studies did not simultaneously consider amyloid and tau pathology as well as WML. A recent cross-sectional study showed that amyloid burden was associated with frailty in older people without dementia, but they did not control for WML and tau pathology (45).

### Dual Tasking

Multivariable analyses that only included one type of brain pathology (controlled for age, sex, education, diagnosis, and comorbidity) showed that CSF Aβ42/40 was significantly associated with the dual-task cost. Our findings are in line with a study by Nielsen et al., which showed a correlation between CSF Aβ42 and dual tasking (*r*_*s*_ = −0.308, *p* < .01) (33). As in the current study, they used a subtraction task and gave no instructions regarding task prioritization. A small PET study (*N* = 27) also indicated that Aβ deposition is associated with dual tasking in cognitively healthy older adults (46). However, the current finding was in contrast with the results by Åhman and colleagues (25), who found no association between CSF Aβ42 and the dual-task cost. This is probably due to methodological differences. Although they had a smaller sample size (*N* = 90 vs. 299 in the current study), it was similar to that of Nielsen and colleagues (*N* = 86) (33). Instead of using a serial subtraction task, Åhman and colleagues used naming animals and reciting months backward (25). Another methodological discrepancy is that they instructed their participants to prioritize walking over the verbal task, which probably made them walk faster (25). The discrepancy in findings signals that the choice of cognitive task is important and/or whether one provides instructions on task prioritization while dual-tasking.

Importantly, our findings in relation to Aβ pathology did not persist when also adjusting for CSF p-tau and WML. CSF p-tau was the only variable that had an independent effect on dual tasking, which applied both when using TUG-Cog and the dual-task cost as dependent variables. These analyses highlight the importance of controlling for other brain pathologies, especially since Aβ and tau pathologies often occur simultaneously as part of AD. The present results are in agreement with a previous study that found a significant correlation between CSF p-tau and the dual-task cost, that is, in relation to mobility and when using a subtraction task (33). Together, these results indicate that tau pathology is associated with decreased dual-task performance, which is in agreement with recent studies showing that tau pathology is more associated with cognitive impairment than WMLs or Aβ pathology (12,19,20). In other words, this study suggests that tau pathology also has an independent effect on cognitive-motor interference. That is, tau pathology worsens mobility performance while doing a simultaneous cognitive task. The effects of dual tasking depend on the type and amount of executive function involvement, and those with amnestic MCI have higher dual-task costs than those with nonamnestic MCI (47,48). A systematic review that addressed neural correlates of cognitive-motor interference highlighted that a direct comparison between studies was difficult. The prefrontal cortex as well as parietal regions seemed to play an important role in dual tasking (30). The strongest effects for tau pathology on cognition seem to be localized in the lateral and medial parietal cortex and lateral temporal cortex (12).

Despite that we controlled for diagnosis (ie, MCI) in multivariable analyses that included the total sample, the independent effect of tau pathology on dual tasking was only prevalent in the MCI group. This might reflect that the variables had a higher variability in the MCI group, which makes it easier to detect an effect. On the other hand, this could be taken as further support for that tau pathology plays a role in dual-task performance, and that dual-task assessment is of importance in relation to people at risk of developing cognitive decline. It needs to be noted that our findings of dual tasking are only valid when assessing mobility in combination with using a subtraction task.

To the best of our knowledge, this is the first study that simultaneously take into account WML as well as Aβ- and tau pathology in relation to dual tasking in older people without dementia. Our findings have several implications. In the total sample, tau pathology was independently associated with dual tasking irrespectively if reporting raw data in relation to TUG-Cog or when calculating the dual-task cost. However, using the dual-task cost instead of the raw data of TUG-Cog might be more specific in relation to AD pathology as indicated by our simple regression analyses (Supplementary Material S1). The volume of WML was then significantly associated with TUG-Cog, which was not the case for the dual-task cost. Both CSF p-tau and amyloid pathology were significantly associated with dual-task cost, which was also the case when adjusting for age, sex, education diagnosis, and comorbidity. In other words, the dual-task cost might be less sensitive to WML and more specific to AD pathology than using the raw data of TUG-Cog. On the other hand, when adjusting for brain pathologies, only p-tau showed an independent effect on the dual-task cost; these findings reflect the importance of taking into account different brain pathologies. All considered, since tau pathology is of relevance for cognitive decline and AD pathology, dual tasking that includes a subtraction task seems important to include in the assessment battery. Moreover, the dual-task cost seems valuable to report. A recent consensus statement by the Canadian Consortium on Neurodegeneration in Aging did in fact include dual tasking in the recommended minimum-battery of tests (49).

### Balance

In the total sample, multivariable analyses showed that CSF Aβ42/40 was independently associated with dynamic balance in older people without dementia. A prior longitudinal PET study showed that higher Aβ burden in putamen was associated with decreased standing (static) balance time in cognitively healthy older adults (17). The current study addressed instead dynamic balance, which is required by most daily activities and transfers. The Figure-of-eight intends to measure dynamic balance (37,38), but one could argue that the timed performance purely mimics walking with a narrow base of support. On the other hand, walking per se challenges balance; this favors including walking when addressing balance control. Balance is indeed a complex construct to assess, and there are numerous clinical instruments available as well as the possibility of using objective measures of postural sway. Interestingly, when using two groups (cognitively unimpaired/MCI), multivariable analyses showed no independent effect of CSF Aβ42/40 on balance; the *p* value was however .073 in the cognitively unimpaired group, whereas it was .207 in the MCI group. The trend in cognitively unimpaired probably reflects a loss of power when compared with the analysis in the total sample. On the other hand, it may suggest that balance impairments are an early sign of amyloid pathology. This is in line with studies which showed that amyloid burden seems to be associated with a decreased time to first fall in preclinical AD (50), and that an abnormal CSF Aβ42/40 ratio is common in hip fracture patients without dementia (51). Further studies are needed that investigate whether amyloid pathology is independently associated with balance impairments and falls in older nondemented people.

### Additional Methodological Considerations

A major strength of the current study is that we simultaneously addressed different brain pathologies by using multivariable analyses in relation to mobility, dual tasking and balance. This approach enabled us to study independent effects. Furthermore, we also included both cognitively unimpaired and MCI, and adjusted for diagnostic group to show that the effect was independent of cognitive status, in addition to independent of age, sex, education, and comorbidity. A limitation is the cross-sectional design that does not allow us to draw conclusions about causality of the observed observations. Replicating studies are needed, in order to support or refute the present findings. Future studies may also consider the effects of α-synuclein and TDP-43 pathology, but there are yet no reliable biomarkers for detection of these pathologies in vivo. The current study focused on the total volume of WML, but analyses of different brain regions would have provided a deeper understanding, which also applies in relation to amyloid and tau pathology.

This study focused on mobility, dual tasking, and balance, but there might be other motor aspects of interest. For example, turning ability is considered to be more complex than walking per se (52,53), and it would be interesting in future studies to relate turning to different brain pathologies. Moreover, it was recently stressed that future research on dual tasking should explicitly focus on turning (54). Studies that focus on turning might benefit from also addressing parkinsonian motor symptoms, as Parkinson’s disease is associated with decreased turning performance (55,56).

## Conclusions

In older people without dementia, common brain pathologies have different effects on motor-related aspects where WML are independently associated with mobility, tau pathology has the strongest effect on dual tasking and amyloid pathology seems to be independently associated with balance. The effects on mobility and dual tasking predominated in the group with MCI, whereas this was not the case for amyloid pathology in relation to balance. Although these novel findings need to be confirmed in longitudinal studies, they suggest that different brain pathologies have different effects on mobility, balance, and dual tasking.

## Supplementary Material

glaa143_suppl_Supplementary_Table

## Data Availability

Anonymized data will be shared by request from a qualified academic investigator for the sole purpose of replicating procedures and results presented in the article and as long as data transfer is in agreement with EU legislation on the general data protection regulation and decisions by the Ethical Review Board of Sweden and Region Skåne.

## Impact Statement

What was already known on this topic: Previous PET studies have shown an association between Aβ burden and decreased gait performance in older people without dementia, but none of these simultaneously addressed WML and tau pathology. WML have been associated with decreased mobility (ie, TUG), whereas PET studies that focused on Aβ and mobility have shown conflicting results. WML have also been associated with balance impairments and falls, but there is limited knowledge in relation to Aβ- and tau pathology. The latter applies also for dual-task performance.

What this study adds: This study provides novel knowledge by using multivariable analyses that take into account WML as well as markers of Aβ- and tau pathology in relation to mobility, dual tasking, and balance performance. Importantly, we also controlled for age, sex, education, diagnosis (MCI), and comorbidity. Our main findings suggest that WML volume is independently associated with decreased mobility, whereas tau pathology is independently associated with worse dual-task performance (ie, in relation to mobility and when adding a cognitive subtraction task). Moreover, our results indicate that Aβ pathology may be of importance for dynamic balance in older people without dementia.
