# Supplementary material for: The Effects of Tau, Amyloid, and White Matter Lesions on Mobility, Dual Tasking, and Balance in Older People
Source: J Gerontol A Biol Sci Med Sci. 2020 Jun 7;76(4):683–91. doi: 10.1093/gerona/glaa143 (PMC8011701; doi:10.1093/gerona/glaa143)
Supplement: glaa143_suppl_Supplementary_Table [file glaa143_suppl_supplementary_table.docx]

**Supplementary Material 1.** Simple regression analyses (total sample); dependent variables are mobility, dual tasking and balance

|  | **Mobility** | **Dual tasking** | | **Balance** |
| --- | --- | --- | --- | --- |
|  | **TUG** | **TUG-Cog** | **Dual task cost** | **Figure-of-eight** |
| Independent variables^1^ | B (95 % CI)  p-value | B (95 % CI)  p-value | B (95 % CI)  p-value | B (95 % CI)  p-value |
| Age, years | 0.079 (-0.001, 0.159)  0.053 | 0.428 (0.031, 0.823)  **0.034** | -2.40 (-5.46, 0.661)  0.234 | 0.077 (-0.142, 0.295)  0.490 |
| Sex (1=woman) | 0.453 (-0.442, 1.35)  0.320 | 3.10 (-1.31, 7.50)  0.167 | -20.6 (-54.6, 13.4)  0.234 | 1.25 (-1.17, 3.68)  0.310 |
| Education, years | -0.111 (-0.233, 0.012)  0.077 | -0.386 (-1.04, 0.269)  0.247 | 1.53 (-3.61, 6.66)  0.559 | -0.574 (-0.940, -0.209)  **0.002** |
| Sample (1=MCI) | 1.51 (0.612, 2.40)  **0.001** | 8.95 (4.57, 13.3)  **<0.001** | -55.1 (-89.2, -20.9)  **0.002** | 3.95 (1.52, 6.37)  **0.002** |
| Stroke (1=yes) | 0.167 (-1.39, 1.72)  0.833 | 6.23 (-1.55, 14.0)  0.116 | -39.0 (-99.1, 21.0)  0.202 | 2.88 (-1.28, 7.05)  0.174 |
| Diabetes (1=yes) | 0.908 (0.531, 2.35)  0.215 | 0.702 (-6.73, 8.14)  0.853 | 5.07 (-52.3, 62.4)  0.862 | 2.79 (-1.14, 6.71)  0.163 |
| Heart disease (1=yes) | 0.174 (-1.17, 1.51)  0.256 | 2.03 (-4.54, 8.61)  0.544 | 9.08 (-59.9, 41.7)  0.725 | 1.28 (-2.32, 4.88)  0.484 |
| WML volume, mL | 0.060 (0.041, 0.080)  **<0.001** | 0.188 (0.097, 0.279)  **<0.001** | -0.596 (-1.30, 0.112)  0.098 | 0.066 (0.007, 0.125)  **0.029** |
| P-tau, ng/ L | -0.004 (-0.023, 0.015)  0.666 | 0.192 (0.099, 0.284)  **<0.001** | -1.62 (-2.34, -0.890)  **<0.001** | 0.059 (0.006, 0.112)  **0.029** |
| CSF Aβ42/40  (1=abnormal) | 0.717 (-0.220, 1.66)  0.133 | 8.27 (3.70, 12.8)  **<0.001** | -61.0 (-96.7, -25.2)  **0.001** | 4.74 (2.15, 7.32)  **<0.001** |

Notes: TUG=Timed Up & Go; TUG-Cog=TUG with concurrent subtraction task; WML=White matter lesions; P-tau=phosphorylated tau; CSF=cerebrospinal fluid; Aβ=β amyloid. Significant p-values are bolded. For dichotomous independent variables, 0=reference category. TUG and the Figure-of-eight are timed (seconds). Dual task cost (%): ([TUG comfortable – TUG-Cog]/TUG comfortable) × 100.

Each simple linear regression analysis quantifies the relationship between just one independent variable (hence "simple") and one dependent variable.

^1^ Some of these variables are used as adjusting factors (age, sex, education, diagnosis, and comorbidity) in multivariable analyses (Tables 2-5).
